# Is It Possible to Bypass the Block in Cerebral Glucose Uptake Following TBI with Exogenous Lactate?

**DOI:** 10.3390/metabo16100800

**Published:** 2026-10-21

**Authors:** George A. Brooks

**Affiliations:** Integrative Biology, University of California, Berkeley, CA 94720, USA; gbrooks@berkeley.edu; Tel.: +1-510-642-2861

**Keywords:** brain fuel, alternative energy source, traumatic brain injury, energy substrate competition, crystalloids, colloids, nutrition, cognition, injury, recovery

## Abstract

**Highlights:**

**What are the main findings?**

- Endogenously produced and exogenously provided lactate is brain fuel.
- The hypothesis that exogenous lactate represents a means to supply a cerebral energy substrate when dietary CHO consumption is limited, and cerebral glucose uptake and use are impaired after brain injury is explored.

**What are the implications of the main findings?**

- Lactate can improve cognition in healthy, uninjured persons.
- Existing vehicles to supply brain fuel are identified, and the possibilities for finding other brain glucose alternatives are discussed.

**Abstract:**

**Background/Objectives**: Foundational studies on brain carbohydrate metabolism are reviewed. More recent results on the unique functionality of lactate and the possibility of augmenting cerebral carbohydrate (CHO) uptake with exogenous lactate to supplement glucose for brain fueling are presented. This review of the physiological evidence and preliminary clinical observations on cerebral lactate metabolism is intended to spur clinical trials leading to improved patient outcomes following brain injury. **Methods**: While glucose is widely recognized as the major brain carbohydrate carbon source, the pathway of degradation involves conversion to lactate that occurs under fully oxygenated conditions. The brain is similar to other tissues in that various energy sources compete for use. For the brain, the alternatives are simpler, being restricted to glucose, lactate and ketone bodies. Although the cerebral metabolic rate of glucose use is diminished following TBI, this limitation may be bypassed in whole or part by supplying lactate as a supplemental brain energy source because uptake is undiminished following TBI. **Results**: The recognition of preferential lactate over glucose use in other tissues and the realization that exogenous lactate may be used to support brain function, normally and in persons following TBI, may be used to our advantage. **Conclusions**: The healthy brain is carbohydrate dependent. In healthy individuals and following injury, the capacity to augment lactate over glucose use provides an opportunity to support brain function. The hypothesis of using exogenous lactate is presented as a promising metabolic strategy that requires further controlled clinical evaluation.

## 1. Introduction

The intent of this paper is to trace a path of discovery of carbohydrate needs of the injured brain that might generate a working hypothesis that lactate supplementation might improve outcomes following traumatic brain injury (TBI). Accordingly, in part, this manuscript mainly provides the broader context for the importance of the Astrocyte-Neuron Lactate Shuttle (ANLS). As reviewed recently by Gerald Dienel [1] a series of landmark studies established glucose as a primary energy substrate for the brain. In 1929 Himwich and Nahum measured arteriovenous (a − v) differences across the brains of dogs and observed respiratory (combustion) quotients [RQ = (v − a)CO_2_/(a − v)O_2_] of 1.00, indicating carbohydrate (CHO) combustion [where a and v represent arterial and venous, respectively] [2]. Subsequently in 1942, Gibbs et al. [3] confirmed high CHO combustion in adult human brains and observed net glucose uptake [positive (a − v)] and net lactate release ([positive (v − a)] from the brain. These findings established that glucose was the major oxidative energy source for the brain. Subsequently, in 1948, Ketty and Schmidt [4] developed a method to determine cerebral blood flow (CBF), which allowed for the calculation of cerebral metabolic rate, i.e., the mass of glucose consumed by the brain [CMR = (Glucose (a − v) × CBF)].

More recently the thesis that the human brain depends largely on glucose as an energy substrate was confirmed and extended by Sokoloff and associates [5,6,7,8]. Replication of results from the 1920s and 1940s led to greater understanding of brain function, and the profound importance of discovery included setting the Recommended Daily Allowance (RDA) for dietary carbohydrate (CHO) to be adequate enough to support the needs of a healthy brain [9]. Pioneers in the field also investigated cerebral use of alternative brain substrates, including ketones [10] and lactate [11]. Unfortunately, data for lactate were not definitive because of intra-cerebral production, and use of lactate was not considered, and the ANLS had not been discovered [12,13]. Fortunately, subsequent studies using tracers, arterial–venous difference and blood flow measurements replicated classical conclusions for glucose and ketones and extended results to demonstrate cerebral lactate uptake and shuttling in resting [14,15] and exercising individuals [16,17]. Perhaps most impressive and relevant to the question of brain lactate supplementation with lactate after TBI are results of studies on healthy exercising men. When arterial lactate concentration rose during exercise due to release from working muscle [18] and other tissue beds, such as the integument [19], in part, lactate substituted for glucose as brain fuel [20], just as it does in heart and red skeletal muscle during exercise [16,17,21,22]. These results on cerebral lactate substitution for glucose in healthy resting and exercising individuals led to the hypothesis regarding use of exogenous lactate bypassing the limitation in cerebral glucose uptake following TBI. Also, it is noteworthy that the discovery of energy substrate competition in the brain was followed by two decades of demonstration of the phenomenon in cardiac metabolism by Bing and colleagues [23,24,25]. Still, considering the history of discoveries of the role of lactate in intermediary metabolism the hypothesis that lactate supplementation might rescue an injured brain was deemed unlikely by some [26]. Hence, some of the history needs to be reviewed for an assessment of the idea that lactate is brain fuel [20].

## 2. Evolution of Lactate Shuttle Theory

### 2.1. Lactate Miscast Despite Nobel Origins

For almost a century lactate has been regarded as the product of oxygen-limited metabolism, a metabolic waste product and muscle fatigue agent [27,28]. However, we now know that in tissues studied to date, lactate is produced and disposed of continuously under fully aerobic conditions [29,30,31,32,33,34], including the brain [35]. Under conditions of physical exercise and postprandial nutrition, lactate production and disposal (flux) can exceed that of any endogenous or exogenous lipid, amino acid or protein. The processes of lactate production, exchange, and disposal serve essential functions in biological systems [36,37,38]. Commencing with seminal hypotheses and experimental results, the path to the idea of cerebral lactate supplementation for the treatment of TBI is reviewed.

### 2.2. Cell–Cell Lactate Shuttling and Origin of the Term Lactate Shuttle

In the 1970s and 1980s radiotracer studies yielded significant progress in determining glucose and lactate production and disposal rates in resting and exercising, trained and untrained lab animals [39,40,41,42]. In parallel, muscle fiber typing and lactate accumulation in the blood and muscles of exercise rats was accomplished [43,44]. A problem then was to rationalize disparate results of lactate exceeding glucose flux and oxidation during exercise and lesser lactate concentration in red compared to white muscle fibers or arterial blood of exercised rats. In this context there arose the hypothesis of a Lactate Shuttle in which lactate from fast white fibers is taken up and oxidized in adjacent red fibers of the same muscle bed or arterial blood that contained lactate released from working white muscle fibers. This hypothesis was articulated in 1984 at a meeting of comparative physiologists in Liege and subsequently published [32]. The original hypothesis was successfully confirmed in studies on resting and exercising, untrained and trained human subjects using stable, non-radioactive tracers and arterial–venous difference together with blood flow measurements across resting and working muscle beds [18,45,46,47,48,49]. Results using [3-^13^C]lactate and [6,6-D2]glucose confirmed significant lactate production and disposal via oxidation and gluconeogenesis in resting humans but also greatly increased lactate production and disposal via oxidation (75–80%) and gluconeogenesis (20–25%) during exercise compared to rest. Similar results were obtained on men after training with lower circulating lactate levels attributable to increased lactate clearance, not lesser lactate production [18,50].

#### 2.2.1. The Astrocyte-Neuron Lactate Shuttle

Since inception of lactate shuttling between cells, tissues and organs in 1984 [32], other lactate shuttles have been discovered. Most prominent among these and relevant to this discussion is the Astrocyte-Neuron Lactate Shuttle (ANLS). Results of studies by Pellerin and Magistretti revealed a linkage between lactate shuttling and glutaminergic signaling, was reported in 1994 [12], although naming of the phenomenon came somewhat later [13,51]. Full development of the course of discovery of the ANLS and its importance will be found elsewhere in associated papers contained in this volume.

#### 2.2.2. Other Cell–Cell Lactate Shuttles

Although data are sparce they are sufficient to indicate the presence of other cell–cell lactate shuttles, including sperm [52,53], and adipose [54,55,56].

##### Intracellular Lactate Shuttling

Cytosol-to-Mitochondrial Lactate Shuttling

The above results combined with those on hearts in men using coronary sinus and arterial catheterization [47,57] show lactate flux and disposal between organs and tissues, such as between muscle and the liver and muscle and the heart. Such exchanges were termed Cell–Cell Lactate Shuttles. However, these data as well as studies on muscles using electron [58] and light microscopy [59] on isolated rodent muscles and myocytes in culture [59] and ultimately on isolated tissue mitochondrial preparations [58,60,61,62] reveal the presence of mitochondrial lactate dehydrogenase (LDH) and monocarboxylate transporters (MCTs). Such results indicate the presence of intracellular lactate shuttling [63]. The presence of an Intracellular Lactate Shuttle was most recently confirmed by investigators using hyperpolarized [1-^13^C]pyruvate with ^13^C magnetic resonance spectroscopy (MRS) [64,65,66].

##### The Peroxisomal Lactate Shuttle

Still another example of intracellular lactate shuttling is between cytosol and peroxisomes [67]. This Peroxisomal Lactate Shuttle (PLS) has been little studied, but it is thought to be important in maintaining cellular redox balance during lipid oxidation [36].

### 2.3. The Postprandial Lactate Shuttle

Physical exercise is an obvious time when carbohydrate carbon flow is elevated and has been studied extensively [18,45,46,47,48,49,50]. However, based on observations of hepatic portal vein lactate appearance after glucose gavage [68], and subsequent red muscle glycolysis in rats [69], the postprandial period was hypothesized to be another time of lactate shuttling [70]. Subsequently, using standard oral glucose tolerance tests but with frequent arterialized blood sampling with D2-glucose and [3-^13^C]lactate tracers aboard the distribution of dietary glucose carbon to lactate and subsequent metabolism was described in young [71] and older men and women [72]. Together with extensive studies on postabsorptive individuals, studies on postprandial volunteers demonstrated that lactate is a major vehicle for carbohydrate carbon distribution and metabolism. Thus, the magnitude of lactate shuttling can be extraordinary, but the events are typical and unelated to oxygen insufficiency [38].

## 3. Healthy Brains and Post-Injury Brain Lactate Metabolism

In critical care medicine elevated lactate (lactatemia) has long been taken as an indicator of severity of illness or injury and poor outcome [73,74,75]. How then did lactate treatment find its way into the neurointensive care setting? The advance became possible for several reasons, including observations on animal experimental models as well as healthy and injured humans. Veracity of the hypothesis is that it was independently developed in several centers by researchers and clinicians [14,20,35,45,76,77,78,79,80]. Some of the trail of discovery is detailed here, but in the end, the path led to a logical conclusion about feasibility of augmenting nutritive support to the injured brain via lactate infusion. Initial efforts were attributable to cross-stimulation of observations and ideas from diverse fields.

In terms of cellular studies of brain lactate metabolism initial discoveries were by Schurr and colleagues, who studied rodent brain preparations and observed the concentration dependence of lactate uptake and the preference of lactate over glucose [79,81]. Similarly, at whole-body, tissue and organ levels when the blood lactate concentration rose in exercising humans, or when exogenous lactate was supplied in the vasculature, lactate not only became the major gluconeogenic precursor [21,82,83], but lactate also substituted for glucose as an energy substrate in working skeletal [21,22] and cardiac muscle [57,84,85]. Similarly, when blood lactate rose in exercising men or in response to injury, cerebral lactate uptake increased in a concentration-dependent manner [15], just as it did in working skeletal muscle [21,22]. Also, the infusion of Na^+^-L-lactate has the effect of alkalinizing the blood [86], a result predictable based on previous studies on dogs [87].

In terms of brain recovery from injury there are important results showing improved cognitive function in brain-injured rats given intravenous lactate therapy [88] and rapid increases in lactate monocarboxylate transporter protein expression in rat brains following TBI [89]. These results were strong inducements for hypothesizing that endogenous lactate production and exogenous lactate supplementation could be beneficial to human TBI patients. Feasibility of and enthusiasm for the approach based on rodent experimentation was heightened when exercise-induced lactatemia augmented cerebral CHO uptake and improved cognitive performance in healthy men [16]. Ultimately, the decision to pursue the hypothesis and experiment on TBI patients were vindicated by extant and subsequent studies of cerebral lactate in resting and exercising human subjects [16,17,90].

## 4. Brain Lactate Metabolism in the Setting of Traumatic Brain Injury

To assess the nutritive needs of injured brains and bodies of brain-injured individuals, we used a combination of isotope tracer and cerebral arterial–venous differences and blood flow technologies to determine glucose–lactate interactions across brains of healthy and TBI patients [14,20]. The isotope tracers used were [6,6-^2^H2]glucose, that is, D2–glucose, and [3-^13^C]lactate, given by arm vein infusion along with simultaneous cerebral arterial and jugular bulb blood sampling. Arterial and tissue PO_2_ were monitored continuously to exclude the possibility of a Pasteur Effect (oxygen limitation) on glucose uptake and lactate production. Cerebral blood flow was determined using the ^133^Xenon technique. Data descriptions and testing were conducted in R version 2.15.1. Groups were compared both with the conventional t test and its robust analogue, the Yuen test, because of the presence of nonnormality in the data distributions. Traumatic brain injury (TBI) patients with nonpenetrating brain injuries (*n* = 12) were entered into the study following consent of patients’ legal representatives. Written and informed consent was obtained from control volunteers (*n* = 6). This necessity meant that patients were studied 5.7 ± 2.2 (mean ± SD) days post-injury, during periods when arterial glucose concentration tended to be higher in TBI patients [14,20].

**Figure 1:**
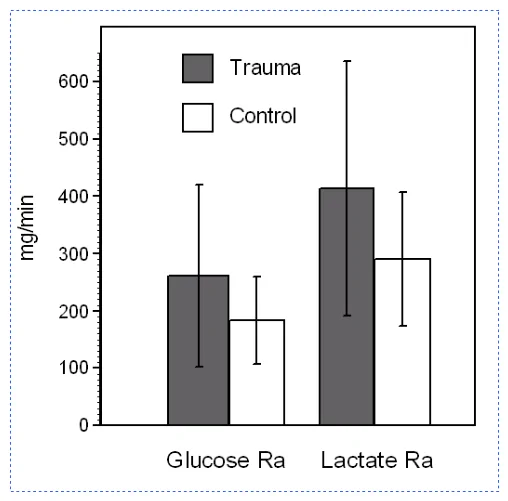
Histograms of whole-body glucose and lactate rates of appearance (Ra) in TBI patients (*n* = 12) and uninjured controls (*n* = 6). Trauma results in large increases in metabolic flux rates to support body and brain. Data from Glenn et al. [14].

**Figure 2:**
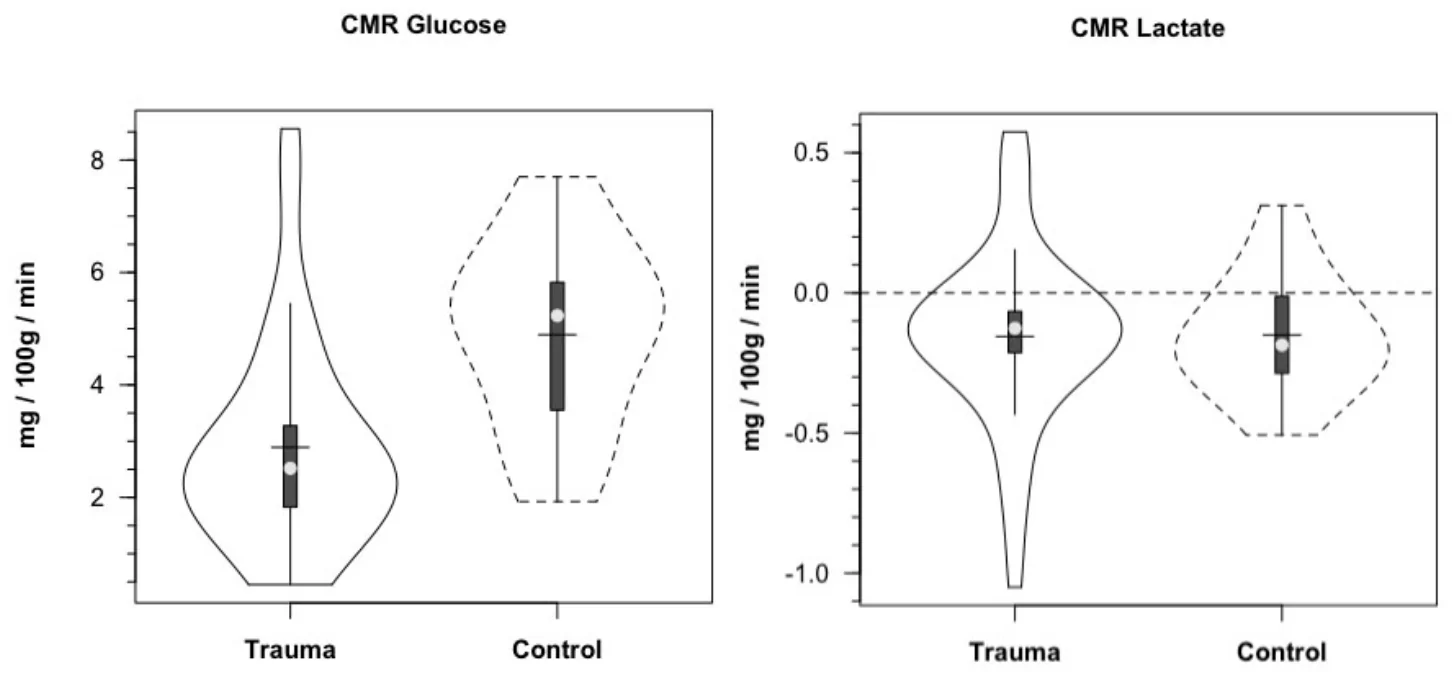
Violin plots of cerebral metabolic rate (CMRgluc, i.e., net uptake) for glucose (**left**) and lactate (**right**) (CMRlac i.e., net release). Solid lines represent traumatic brain injury (TBI) patients (*n* = 12), whereas dashed lines are normal control subjects (*n* = 6). Symbols: median (light circle), mean (short horizontal line), standard deviation (heavy vertical bar), box-plot whisker (thin vertical bar), and a kernel density estimation of the data distribution (replacing the box-plot’s rectangular depiction). From Glenn et al. [20].

**Figure 3:**
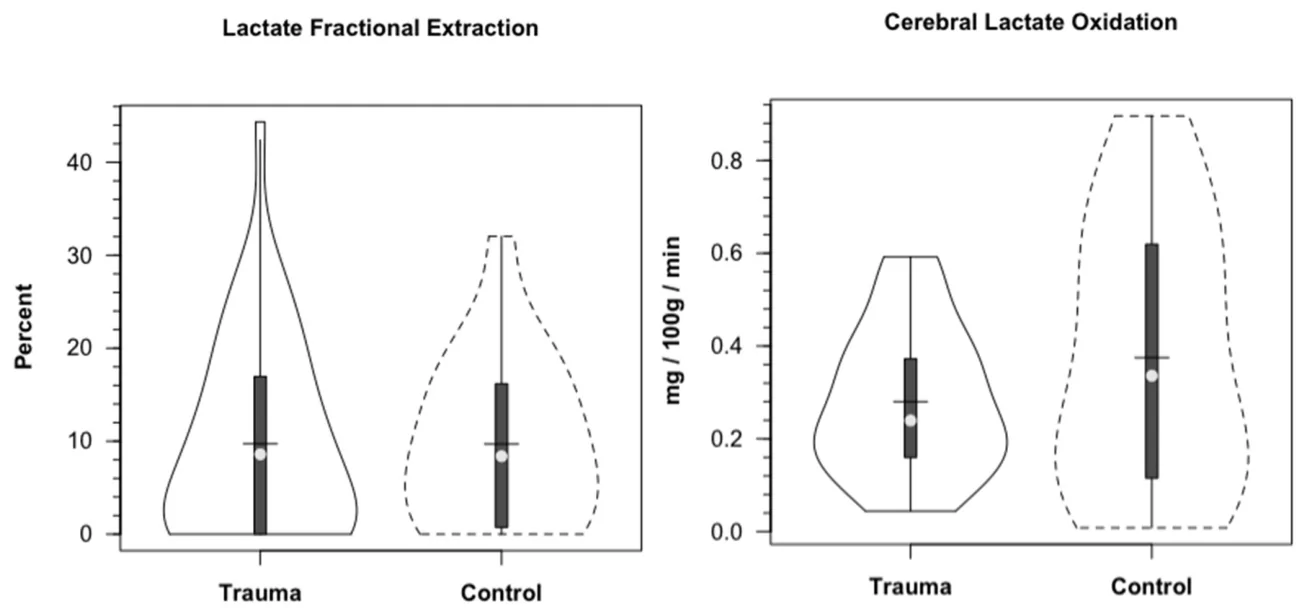
Violin plots of cerebral lactate fractional extraction (**left**) and rate of lactate oxidation (**right**) in TBI patients and uninjured controls. Solid lines represent traumatic brain injury (TBI) patients (*n* = 12), whereas dashed lines are normal control subjects (*n* = 6). Cerebral ability to take up and oxidize (utilize) lactate is undiminished after injury. These results reveal the possibility of supporting cerebral CHO needs with exogenous lactate. Symbols as in Figure 2. From Glenn et al. [20].

Not surprisingly after TBI we observed great mobilization of body resources, mainly lactate but also glucose, to support the brain-injured individual (Figure 1). Hepatic and renal gluconeogenesis (GNG) from lactate was essential to indirectly supply glucose to the injured brain [14]. Yet, despite the corporal response to rally energy support, glucose uptake by the injured brain was significantly depressed (Figure 2(left)). However, as was seen in previous investigations, cerebral metabolic rates for glucose (CMRgluc, i.e., net glucose uptakes) were significantly suppressed following TBI (*p* < 0.001). However, the CMR for glucose was decreased, and the CMR lactate was undiminished following TBI (Figure 2(right)). Furthermore, lactate fractional extraction, an index of cerebral lactate uptake related to systemic lactate supply, approximated 11% in both healthy control subjects and TBI patients (Figure 3(left)). Moreover, the percentages of lactate tracer taken up and released as ^13^CO_2_ into the jugular bulb venous drainage accounted for 92% and 91% for control and TBI subjects, respectively (Figure 3(right)). These results indicate that most cerebral lactate uptake was oxidized following TBI [20].

## 5. Lactate Substituting for Glucose as Brain Fuel: Seeing Is Believing

**Figure 4:**
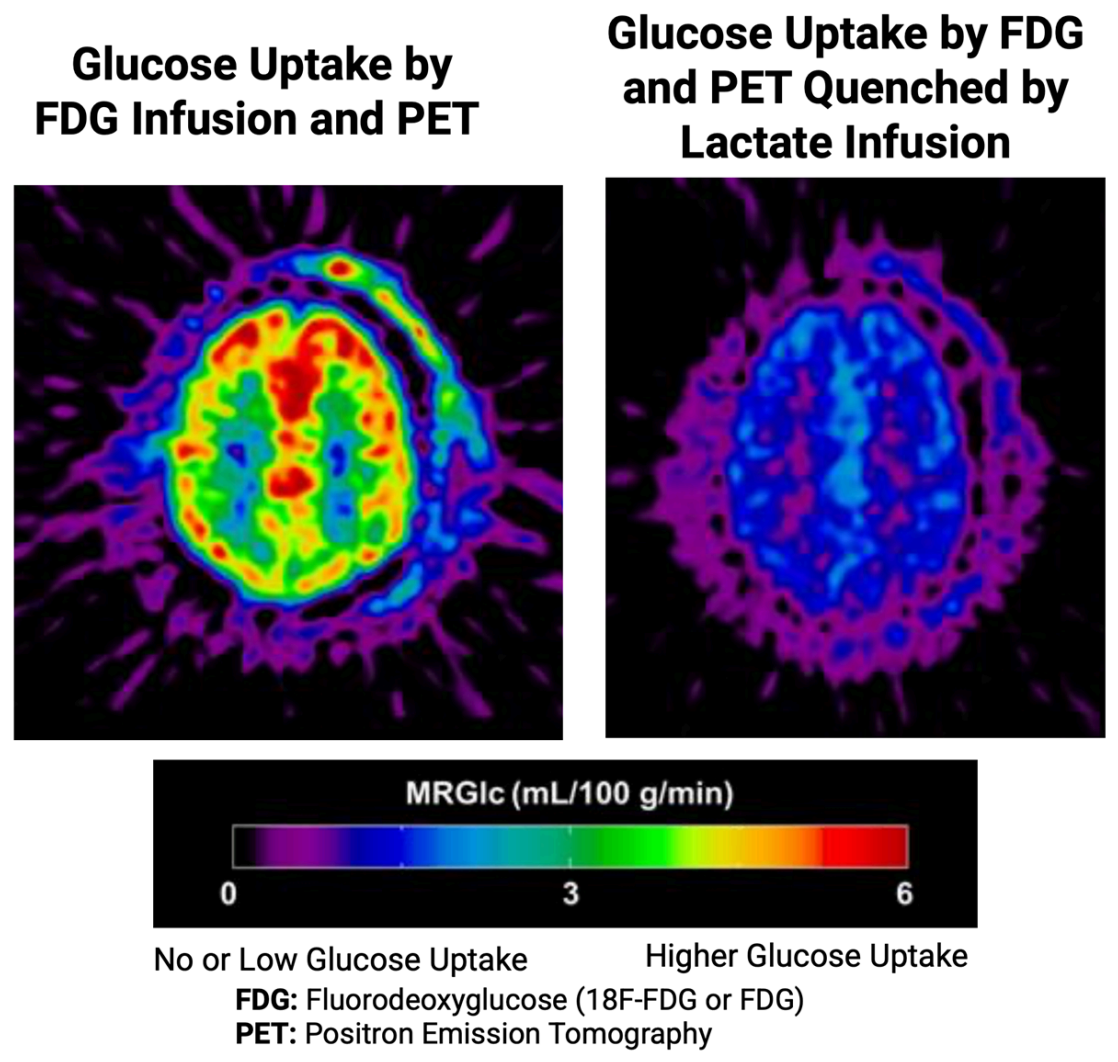
Fluorodeoxyglucose, FDG–positron emission tomography scans (FDG-PET scans) of a traumatic brain injury (TBI) patient (**left**) and (**right**) (the same patient with lactate infusion). Comparison with panels (**left**, **right**) shows exogenous lactate quenching of brain glucose uptake as following infusion of the radioactive glucose analog fluorodeoxyglucose (18F-FDG or FDG) and measurement by Positron Emission Tomography (PET) to detect glucose uptake. Scans courtesy of T.C. Glenn [14,20]. Figure Created in BioRender. Brooks, G. A. (2026) https://BioRender.com/8cjcwc3 (accessed on 11 October 2026).

From the standpoint of traditional thinking, it would have been surprising that at a whole-body level in healthy men exogenously provided lactate would diminish and partially substitute for glucose as well as cause a mild alkalosis, but it does [21,22,86]. More relevant to brain metabolism and TBI is that using the preference for lactate over glucose as brain fuel is illustrated by lactate quenching brain glucose uptake measured by infusion of the radioactive glucose analog fluorodeoxyglucose (^18^F-FDG, or FDG) and measuring cerebral glucose uptake by positron emission tomography (PET) scanning. The preference for the injured brain for lactate over glucose uptake is clearly seen in Figure 4.

## 6. Efficacy of Providing Exogenous Lactate for Nutritive Brain Support Following TBI

**Figure 5:**
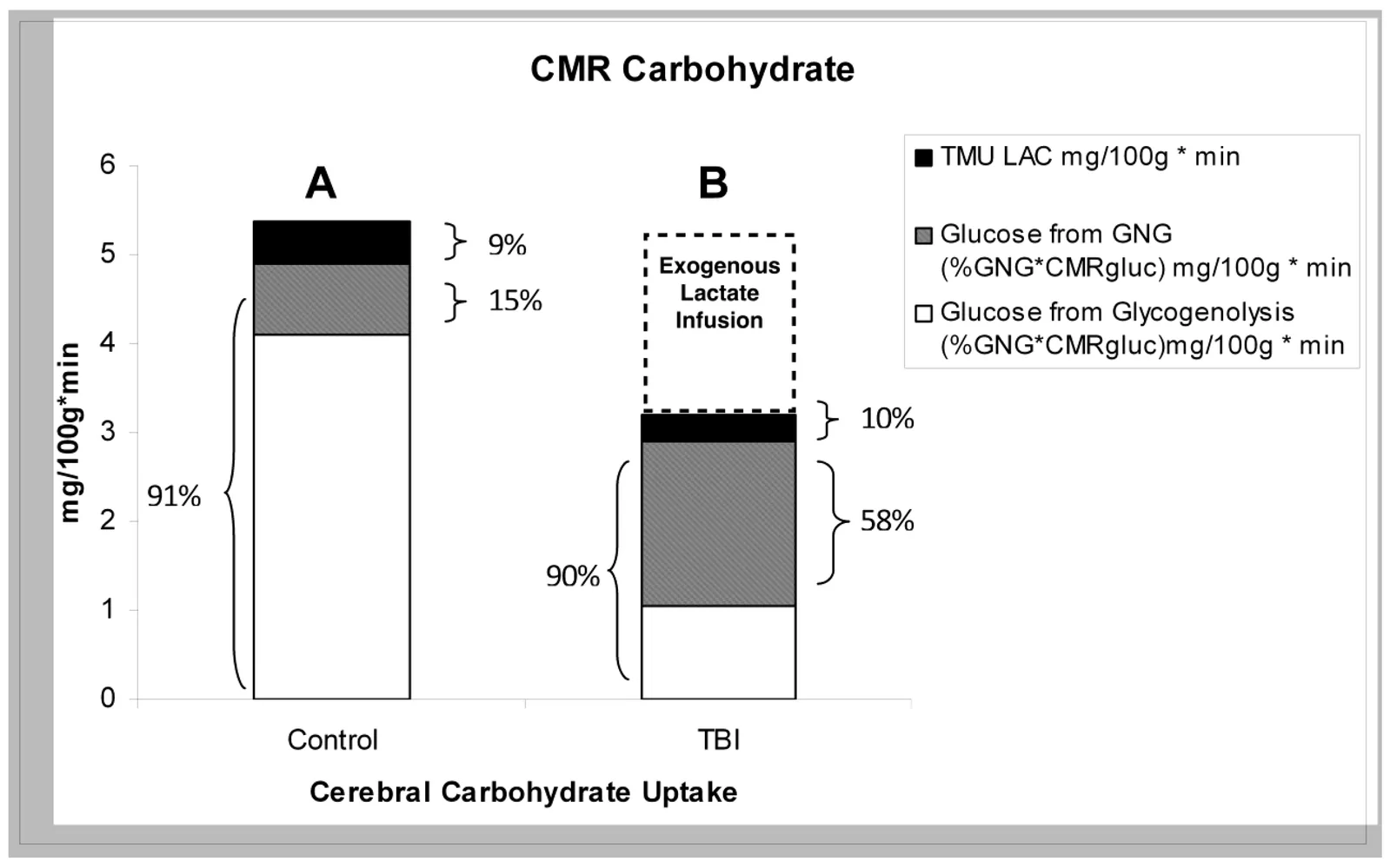
Histograms of the absolute and relative contributions to total cerebral carbohydrate (CHO) from lactate, glucose from gluconeogenesis (GNG), and glucose from hepatic glycogenolysis in healthy control subjects (**left**) and traumatic brain injury (TBI) patients (**right**). Compared with **panel** (**left**), **panel** (**right**) not only shows the decrease in cerebral metabolic rate for glucose (CMRgluc) following TBI but also an increased relative contribution of glucose from GNG to total cerebral CHO uptake after TBI. A comparison of panel (**left**) (healthy controls, *n* = 6) and (**right**) (TBI patients, *n* = 12) shows the large increase in percentage cerebral CHO uptake contributed by lactate, directly or indirectly from GNG, following TBI. Because of the undiminished ability of the injured brain to take up and utilize lactate (Figure 3), the top of panel (**right**) shows the potential for using supplemental lactate to fuel the injured brain. Redrawn from Brooks and Martin [35]. * means times, or multiply.

The results presented in the above data and discussion are summarized in histogram form in Figure 5, where cerebral carbohydrate (CHO) energy is considered to represent glucose-plus-lactate uptake [20]. In healthy controls, glucose accounts for 91% of the total energy substrate. Of that amount 15% was supplied by glucose for gluconeogenesis, and direct lactate uptake approximated 9% (Figure 5(left)). In comparison, the situation in TBI patients was very different (Figure 5(right)). Post-TBI glucose again provided 91% of CHO energy and direct lactate oxidation 10% of cerebral CHO uptake. However, total glucose uptake was severely depressed with most of that supplied by gluconeogenesis from lactate. Consequently, the rationale for attempting to cover the injured brain CHO deficit by supplying exogenous lactate as brain fuel is shown at the top of column 5 right. Simply, because cerebral glucose uptake is limited (Figure 2(left)), while lactate uptake and oxidation are not (Figure 2(right) and Figure 3), the rationale is to bypass the block in glucose flux by augmenting lactate flux. The reason for diminished post-TBI glucose uptake is unknown, perhaps by a redox change at GAP-DH making exogenous glucose, with and without insulin being ineffective in raising cerebral CHO uptake. However, because lactate uptake is not injury limited, the idea is to take advantage of functional brain cell lactate transport proteins (monocarboxylate transporters) and increase cerebral CHO uptake with exogenous L-(+)-lactate. Moreover, using FDG and PET the capacity for the injured brain to preferentially take up lactate is seen (Figure 4). In sum, these observations may be interpreted to mean that lactate supplementation could be a means of increasing brain carbohydrate uptake following TBI.

When considering the idea of exogenous lactate in relation to TBI patients’ doses, algorithms regarding the route of administration, infusion duration, blood lactate targets, monitoring strategy, and patient heterogeneity will need to be established. But, where to start? Fortunately, lactate clamp studies performed on healthy young men may provide guidance [21,22,86]. A concentrated sodium-(L)-lactate solution (≤1000 mOs/L) was infused via a large vein or central line at a rate sufficient to increase arterial [lactate] to 4.0–4.5 mM for a duration of 3 h, OD or BD, depending on patient responses. Because lactate is a gluconeogenic (GNG) precursor as well as a monocarboxylate (MC), depending on patient status and enteral or parenteral nutrition, lactate infusion may cause blood [glucose] to exceed 6 mM. In that case the lactate infusion rate may be reduced while maintaining a 4 mM target or insulin therapy initiated. Lactate infusion to a target may obviate issues related to body size, sex, or time between last meal and injury for initiating exogenous lactate infusion treatment of brain injuries.

Of course, extensive multi-site clinical trials will be necessary to test the hypothesis of improved patient outcomes by lactate supplementation following TBI.

## 7. Metabolite Shuttling and Competition

As noted above, competition among metabolites in human cardiac metabolism was observed in the mid-20th century [24], and iterations of the concept have appeared subsequently in the form of glucose-fatty acid cycle [91] and the crossover concept [92]. Of the latter two concepts the difference is that fatty acid flux dominates over carbohydrates, whereas carbohydrate (CHO) dominates in the latter. In these initial studies of the competition among fat, CHO and amino acid-derived fuels, the role of ketones was also investigated [23]. Subsequently, cerebral use of ketones was also demonstrated [10]. However, in the context of dietary energy sufficiency, a significant role of ketones appears in starvation [93].

Also noted above is that in rats, brain injury results in a rapid increase in lactate transporters, MCTs [89]. Because of similarities in structures of lactate and beta-hydroxy butyrate (β-OHB) it is not surprising that (β-OHB) crosses plasma membranes via lactate transporters (MCTs). Important in this context however is that the Vmax is lower and KM higher for β-OHB compared to lactate [94,95]. Still, it is to be expected that when dietary CHO and energy are inadequate, ketone bodies are produced in the liver, released into the systemic circulation, and are available as an alternative energy fuels in children [96].

Providing an energy substrate to brain-injured individuals requires an understanding of some investigators, such as Plourde et al., who are interested in using β-OHB as an alternative post-injury brain fuel [97]. Even though ketones are the least preferred energy source it is to be appreciated that there is also competition among tissues for energy substrates. Consider a glycogen-depleted TBI patient who is in negative energy, nitrogen and CHO balance due to greater muscle mass and demand or glucose and lactate energy sources; the brain would benefit by access to ketone bodies (β-OHB and aceto-acetate) as energy and pentose pathway sources [97]. However, despite enthusiasm for ketones as fuel energy sources, in this work emphasis is placed on lactate as a brain energy source, owing to far greater availability research on healthy and brain-injured humans compared to rodent study models.

## 8. Multiple Cerebral Effects of Lactate Supplementation

In this work emphasis has been placed on the role of lactate as an energy source, but there are other potential beneficial effects. In health lactate is the major gluconeogenic precursor [83], but such is also the case following TBI [14] (Figure 3). And although CMR of glucose is limited after injury (Figure 2), the stress of bodily injury increases whole-body glycolysis and lactate production (Figure 1). These compensatory strain responses serve to minimize the stress of injury by making glucose available. Furthermore, lactate has an effect of stimulating blood flow [36], and an effect of exogenous, hypertonic lactate infusion on increasing cerebral blood flow (CBF) has been observed by Carteron et al. [78]. In addition to increasing glucose and lactate availability, increased CBF is thought to minimize proinflammatory events and swelling post-TBI [35]. Potential effects of the lactate anion carrier, sodium ion, are noted below.

## 9. Lactate Signaling

Historically the third component of Lactate Shuttle Theory has been that of lactate as a metabolic signal [98,99]. Lactate signals by various means, including changing redox, ROS generation [100], allosteric binding via HCAR [54], and histone lactylation [101,102]. Among the recently discovered roles of lactate are those in cells [103], embryonic development [104] and differentiation [105]. Seemingly, additional lactate signaling functions are being discovered monthly. Among these perhaps the most important has been the discovery of the Astrocyte-Neuron Lactate Shuttle, the original function of which is glutaminergic signaling. As noted above, this finding is described in accompanying papers.

## 10. Vehicles for Lactate Delivery

Inorganic Lactate Salts: To date sodium L-(+)-lactate has been used to supplement cerebral CHO uptake following TBI [77,78,106,107,108,109,110]. The lactate infusate concentrations used have been as high as half molar Na^+^-L-(+)-lactate (1000 mOsm/L), which is at the upper safe limit for the delivery of hypertonic solutions [78]. Because sodium ion-induced hypertonicity experiments using half molar Na^+^-lactate are typical, in the field of cardiac resuscitation 24 h of infusion improved cardiac performance without ill effects [111]. However, other than sodium ion, other means to deliver lactate anion are possible [112,113].

Amino Acid Carriers: Infusing hypertonic sodium cation solutions causes a mild alkalosis and raises the possibility of upsetting fluid-electrolyte balance. Hence, it will be prudent to consider renal function that may or may not be intact following body trauma that results in TBI and other injuries. Although apparently not investigated in the realm of brain injury, in the field of sports nutrition organic lactate carrier molecules have been used. These include amino acid zwitter ions, such arginine lactate anion carriers [114,115]. Functionality of the compound may in part be attributable to arginine, which is a nitric oxide vasodilator precursor. Arginyl lactate is Generally Accepted as Safe (GRAS) by the US Food and Drug Administration (FDA) and at present could be administered enterally to brain-injured persons.

## 11. Discussion

**Figure 6:**
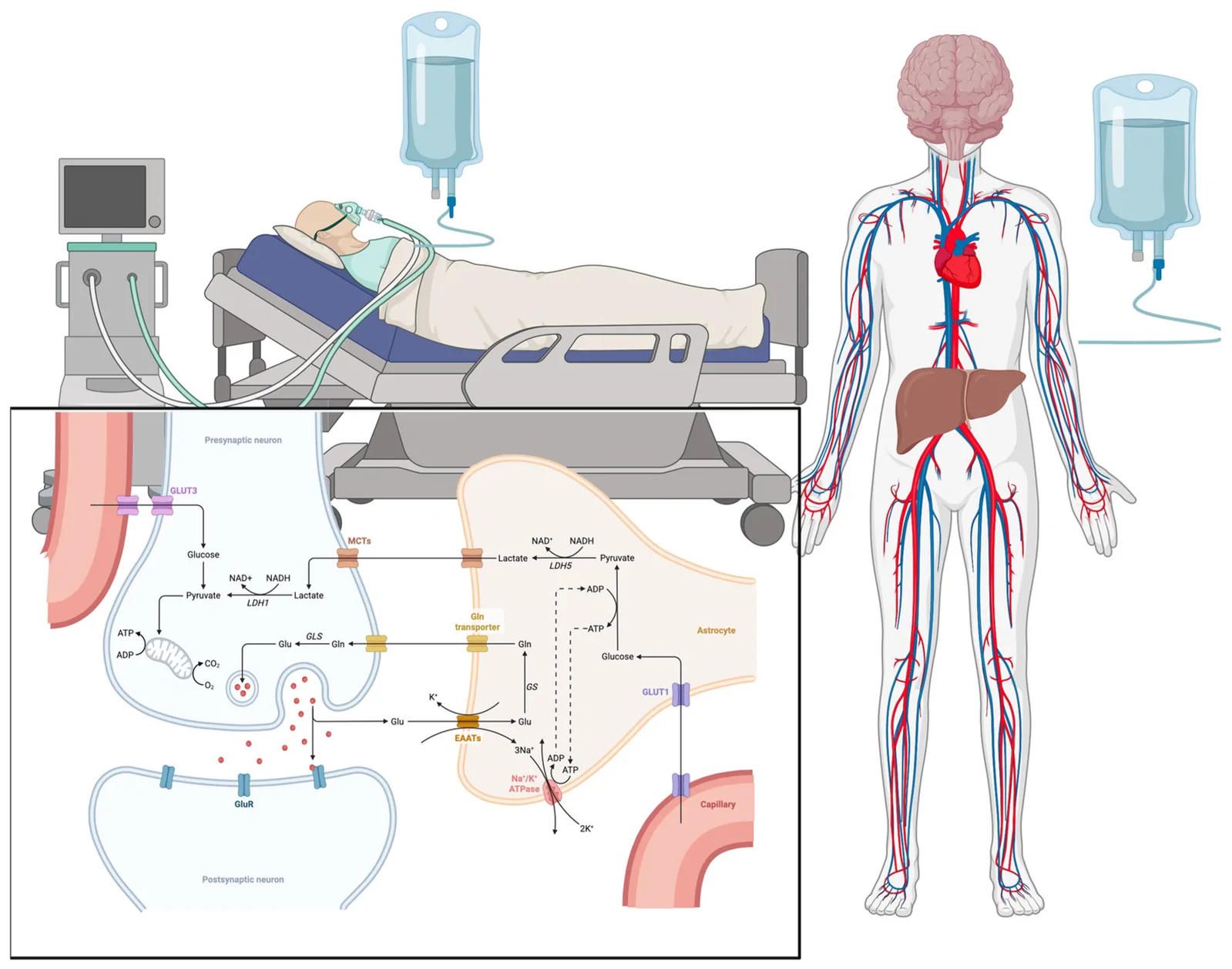
Cartoon showing motivation for supplying exogenous lactate to a brain-injured person is illustrated. The brain is nourished by endogenous and exogenous glucose and lactate. For an unconscious brain-injured person, a sympathetic response mobilizes liver and skeletal muscle glycogen. Lactate released because of glycogenolysis and glycolysis into the sympathetic circulation can cross the blood brain barrier (BBB) via monocarboxylate lactate transport proteins (MCTs) and directly supply nutritive support to the injured person. Also, exogenous lactate supports hepatic and renal gluconeogenesis, which supports blood glucose homeostasis. Glucose crosses the BBB via the non-insulin regulatable glucose transporter (GLUT1). Subsequently as a result of glycolysis in astrocytes, lactate is released into the interstitial space and supports energy homeostasis in neurons and processes such as glutaminergic signaling [13]. Brooks, G. A. (2026) (https://BioRender.com/5rqqm42 (accessed on 11 October 2026)) is licensed under CC BY 4.0.

Given traditional misunderstanding about lactate and its functions in physiology and metabolism and the wide use of Lactated Ringers as a crystalloid to support plasma volume in illnesses and after injuries, it might be understandable that some would propose ketones as another alternative fuel. Support or the idea of providing cerebral support comes from basic studies of brain metabolism [10] as well as the use of a ketogenic diet for children with epilepsy [116]. However, it is to be realized that ketones cross the brain barrier by MCTs, but these transporters preferentially transport lactate and pyruvate [94,95]. Pyruvate infusion could also be considered as a means for parenteral or enteral compounds to support cerebral nutritive support, but pyruvate is rapidly converted to lactate during one circulatory passage, owing to the abundance of MCTs and LDH in erythrocytes and lung parenchyma [117]. Hence, clinical trials will be necessary to assess the types and regionalities of injuries when monocarboxylate supplementation would improve outcomes after bran injuries and establish the use of lactate or other alternative fuels for the efficacious management of brain-injured persons. A depiction of how lactate infusion and monitoring that someday might become part of the arsenal of procedures and equipment to manage brain injury is depicted in Figure 6.

## 12. Summary

The history of studies on lactate shuttling commenced with studies of resting and exercising lab animals [39,41,42,118]. The key role of lactate shuttling in postabsorptive resting and exercising humans was subsequently described [18,45,46,47]. During exercise extra- and intracellular lactate shuttling served to exchange carbon for oxidative phosphorylation, gluconeogenesis and cell signaling as seen in resting and exercising postabsorptive individuals [113,119]. Subsequently, the presence of other lactate shuttles was recognized. Importantly, at the level of basic science, the presence of an Astrocyte-Neuron Lactate Shuttle was recognized. That discovery has its own history as eloquently described by others [12,13]. Importantly, in terms of brain fueling cerebral lactate uptake in exercising humans was shown to be concentration dependent [16,17,90]. Despite a century of bias [27,28,120], inevitably, it was recognized that lactate played a major role in postprandial carbon flux [70,71,72].

Admittedly, the positing of a positive roles for lactate as a vehicle for carbohydrate carbon delivery during exercise and following CHO nutrition has been controversial [70]. Similarly, positing functional roles for brain lactate [12,81,121,122,123] has been duly met with controversy [124]. Hence, while at present it is reasonable to posit that lactate supplementation could be used to provide nutritive support to the injured brain by bypassing the block in glucose uptake [35], to become standard of care the hypothesis needs to be rigorously tested in multiple centers of excellence [123,124]. The hypothesis of using exogenous lactate is presented as a promising metabolic strategy that requires further controlled clinical evaluation.

## Data Availability

Published articles are available online. Datasets are posted in primary references and supplemental archives online. Sharing other raw data supporting the conclusions of primary articles may be considered.
